# Application of ultrasound multimodal imaging in the prediction of cervical tuberculous lymphadenitis rupture

**DOI:** 10.1017/S0950268824000153

**Published:** 2024-01-30

**Authors:** Dan Zhao, Na Feng, Ning He, Jie Chu, Yaqin Shao, Wenzhi Zhang

**Affiliations:** Department of Ultrasound, Hangzhou Red Cross Hospital, Tuberculosis Diagnostic and Treatment Center of Zhejiang Province, Hangzhou, China

**Keywords:** cervical tuberculous lymphadenitis, rupture, prediction, ultrasound multimodal imaging, Ultrasonic features

## Abstract

Lymph node tuberculosis is particularly common in regions with a high tuberculosis burden, and it has a great risk of rupture. This study aims to investigate the utility of ultrasound multimodal imaging in predicting the rupture of cervical tuberculous lymphadenitis (CTL). 128 patients with unruptured CTL confirmed by pathology or laboratory tests were included. Various ultrasonic image features, including long-to-short-axis ratio (L/S), margin, internal echotexture, coarse calcification, Color Doppler Flow Imaging (CDFI), perinodal echogenicity, elastography score, and non-enhanced area proportion in contrast-enhanced ultrasound (CEUS), were analyzed to determine their predictive value for CTL rupture within a one-year follow-up period. As a result, L/S (*P* < 0.001), margin (*P* < 0.001), internal echotexture (*P* < 0.001), coarse calcification (*P* < 0.001), perinodal echogenicity (*P* < 0.001), and the area of non-enhancement in CEUS (*P* < 0.001) were identified as significant imaging features for predicting CTL rupture. The prognostic prediction showed a sensitivity of 89.29%, specificity of 100%, accuracy of 95.31%, respectively. Imaging findings such as L/S < 2, unclear margin, heterogeneous internal echotexture, perinodal echogenicity changed, and non-enhancement area in CEUS > 1/2, are indicative of CTL rupture, while coarse calcification in the lymph nodes is associated with a favorable prognosis.

## Introduction

Tuberculosis (TB) remains a major global health concern, with millions of people affected by the disease and a significant number of deaths each year [1]. Among the different forms of TB, lymph node TB is particularly common in regions with a high TB burden, such as China [2, 3].

Cervical tuberculous lymphadenitis (CTL) can cause considerable discomfort and pain to patients due to its chronic nature, difficulty in achieving a complete cure, and the risk of rupture [4, 5]. Ruptured lymph node tuberculosis can lead to the formation of sinus tracts that take a long time to heal [3]. In some cases, the pyogenic fluids ruptured from the CTL can spread to adjacent organs or major blood vessels, leading to organ dysfunction and posing a serious threat to life [3, 6]. While systemic chemotherapy is effective for most patients, some individuals may experience poor response due to drug resistance or other factors [7–9]. Surgical removal of the affected lymph nodes can prevent extensive ulceration and sinus tract formation, but it carries risks and complications, such as poor wound healing and scar tissue formation, limiting its application [2–6]. Therefore, there is an urgent need to develop a system that can predict and assess the risk of lymph node tuberculosis rupture, evaluate the effectiveness of chemotherapy, and guide surgical interventions. This study aims to explore the feasibility of predicting CTL rupture using multimodal ultrasound characteristics as indicators. The findings could provide a theoretical basis for evaluating clinical efficacy and determining the optimal timing for surgical intervention.

## Materials and methods

### Patients

This retrospective study included patients with cervical lymphadenopathy who visited our hospital between January 2020 and December 2021 and were subsequently diagnosed with lymph node tuberculosis. The study was approved by the Ethical Committee of Hangzhou Red Cross Hospital (No. 2021-298), and informed consent was obtained from all participants.

The study employed specific inclusion and exclusion criteria to ensure the selection of appropriate participants. The inclusion criteria comprised the following: (1) presence of enlarged lymph nodes in the neck, (2) confirmation of lymph node tuberculosis through pathological or laboratory examination, (3) absence of allergic constitution and ability to tolerate contrast-enhanced ultrasound (CEUS) examination, (4) absence of any other underlying diseases and ability to undergo needle biopsy, and (5) availability of comprehensive imaging data, including conventional ultrasound, elastography, CEUS, and needle biopsy, obtained during the initial diagnosis at Hangzhou Red Cross Hospital. Furthermore, participants needed to give consent for their data to be used in the analysis of this study. Conversely, the exclusion criteria consisted of the following: (1) incomplete data, (2) unclear diagnosis conclusions, (3) occurrence of ulceration during the initial visit, (4) inability to attend follow-up visits for a duration of 1 year, (5) occurrence of lymph node rupture within 15 days after biopsy, and (6) prior receipt of anti-TB treatment before the first visit to our hospital.

Ultimately, the study included a total of 128 cases, comprising 48 males and 80 females, with ages ranging from 19 to 67 years and an average age of 36.22 ± 9.449 years. Participants were followed up for a period of 1 year following the acquisition of complete ultrasound data. The primary end point of the study was the occurrence of lymph node rupture within this 1-year time frame. It is important to note that none of the enrolled cases had received anti-TB treatment prior to their initial diagnosis. However, if necessary, participants were allowed to receive relevant anti-TB treatment within 1 year following the diagnosis.

### Ultrasound examination and core-needle biopsy

**Figure 1.**
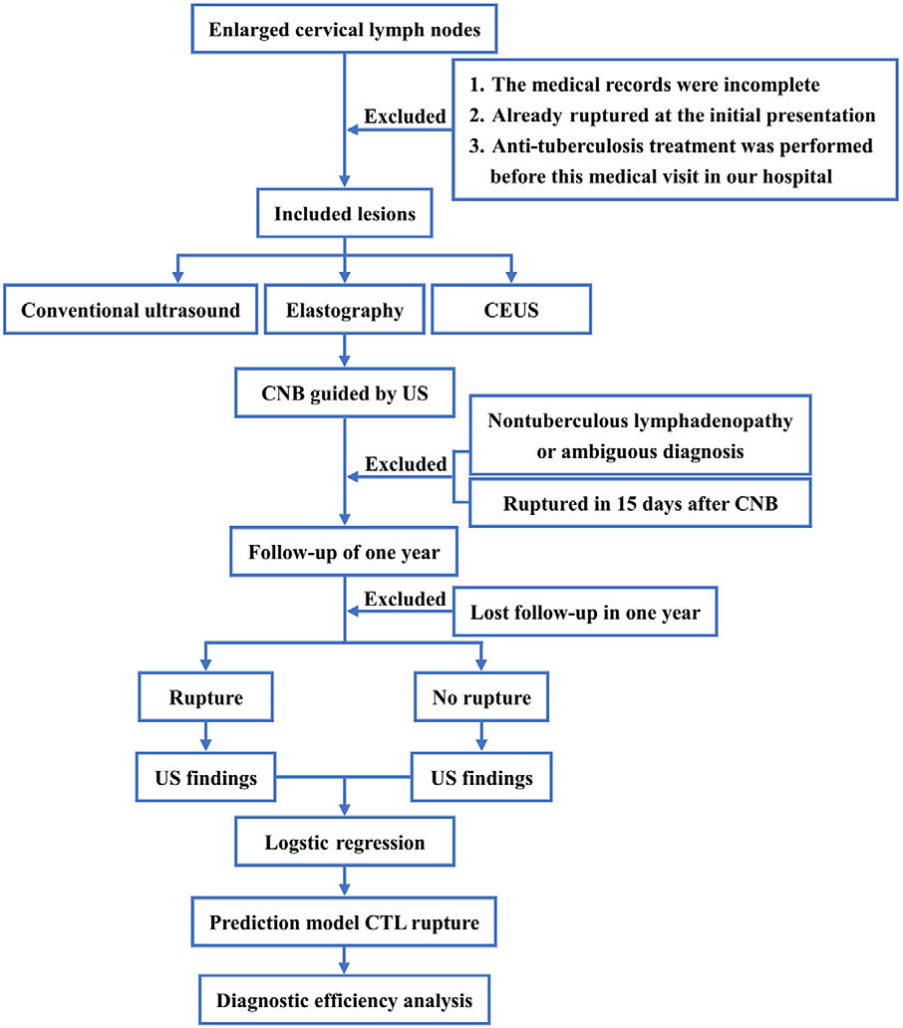
The flowchart.

The diagnosis utilized the Philips iU-Elite ultrasonic diagnostic apparatus (Washington, DC), with the L12-5 broadband linear array probe (frequency 5–12 MHz) used for conventional ultrasonography. Patients were positioned supine with the neck fully exposed, and the largest lymph node was identified through region-by-region examination, with recorded ultrasound signs. Elastography was performed and scored according to the Itoh Elasticity Scale [10]. Subsequently, CEUS was conducted with the L9-3 broadband linear array probe (frequency 3–9 MHz), utilizing pulsed inverse harmonic imaging technology with a low mechanical index (0.06). The contrast agent, SonoVue (Bracco, Milan, Italy), was initially diluted with 5 ml of normal saline, shaken, and then 2.4 ml of the solution was administered via bolus injection through the superficial vein of the elbow, followed by a 10 ml flush of normal saline. The timing of the contrast agent injection was recorded, and the perfusion of the entire lymph node was dynamically observed using the double-amplitude contrast interface. This observation was continuously conducted for 3 minutes, and the entire imaging process was stored. The proportion of the non-enhanced area on the long-axis section of the lymph node with the largest non-enhanced area was analyzed, calculated, and recorded. Two senior radiologists analyzed, evaluated, and documented the entire process. In the event of any disagreement, a third senior radiologist was involved in the discussion to reach a conclusion. The process flow is illustrated in Figure 1.

Following the CEUS examination, a core-needle biopsy was immediately performed under ultrasound guidance using the L12-5 probe. The biopsy specimens were subjected to pathology and laboratory examination, ultimately confirming the diagnosis of lymph node tuberculosis. It was confirmed by acid-fast staining and next-generation sequencing in pathology or by Xpert MTB/RIF in laboratory. The choice of puncture path was determined by two senior radiologists based on the analysis of conventional ultrasound and CEUS. In cases of disagreement, a third senior radiologist was consulted.

### Statistical analysis

Data processing was performed using the SPSS statistical software package (version 23.0; SPSS Inc., Chicago, IL) and MedCalc (version 18.2.1; MedCalc Software Ltd, Ostend, Belgium). The chi-square test and Fisher’s exact test were utilized to assess the differences between the ruptured group and unruptured group for count data. The *t*-test was employed for analyzing measurement data. *P* < 0.05 was considered statistically significant.

Various ultrasound multimodal sonographic features of the lesion, such as the long-to-short-axis ratio (L/S), margin, internal echo, coarse calcification, colour Doppler flow imaging (CDFI), perinodal echogenicity, elasticity score, and proportion of non-enhanced areas on CEUS, along with patient demographics such as age and gender, were subjected to univariate analysis as independent variables. Ultrasonic characteristic variables demonstrating statistical significance were selected and included in the binary logistic multivariate regression analysis. The regression model provided partial regression coefficients (*β*) for each ultrasonic image rupture sign. Receiver operating characteristic (ROC) curves were constructed, and the test level was set at *α* = 0.05.

## Results

Among the 128 cases of CTL, the volume of the lesions was calculated using the ellipsoid model in millilitres, with an average volume of 3.66 ± 1.055 ml. Diagnosis of lymph node tuberculosis was confirmed through pathological or laboratory examination. Based on the occurrence of rupture during the 1-year follow-up period, the cases were divided into two groups: the ruptured group, which consisted of 56 cases (43.75%), and the unruptured group, which comprised 72 cases (56.25%).

### Univariate analysis

Table 1 presents the results of the univariate analysis conducted to identify predictors of TB and ulceration in cervical lymph nodes. The analysis revealed several significant findings. Firstly, the average age of the ruptured group was found to be younger compared to the unruptured group. Additionally, the lesion volume in the ruptured group was observed to be larger than that in the unruptured group, while the cut-off value was 3.73 ml (Youden’s index: 0.7103, sensitivity: 82.14%, specificity: 88.89%, 95% confidence interval [CI]: 0.865 to 0.964).

**Table 1.** Basic characteristics of the patients and ultrasound features of the lesions

	Ruptured (*n* = 56)	Unruptured (*n* = 72)	*t*/*x* ^2^	P
Mean age (years)(M ± SD)	34.02 ± 8.223	37.93 ± 10.027	−2.366	0.020*
Gender (female/male)	36/20	44/28	0.135	0.713
Volume of the lesion(ml)	4.48 ± 0.903	3.01 ± 0.633	10.813	0.000*
L/S			33.633	0.000*
<2	46	22		
≥2	10	50		
Margin			77.432	0.000*
Clear	7	65		
Unclear	49	7		
Echotexture			18.807	0.000*
Homogeneous	10	40		
Heterogeneous	46	32		
Coarse calcification			29.958	0.000*
Absence	55	40		
Presence	1	32		
Internal CDFI			0.749	0.387
Hypovascular	51	62		
Hypervascular	5	10		
Perinodal echogenicity			74.598	0.000*
Not changed	11	68		
Changed	45	4		
Score of elastography			0.999	0.133
2	23	37		
3	32	30		
4	1	5		
Non-enhanced area proportion			55.342	0.000*
≤1/2	5	54		
>1/2	51	18		

CDFI, colour Doppler flow imaging; LN, lymph node; L/S, long-to-short-axis ratio.

* Statistically significant difference.

Regarding ultrasound signs, the presence of L/S <2, unclear margin, heterogeneous internal echotexture, perinodal echogenicity changed, and a proportion of non-enhanced area in CEUS >1/2 were found to be significantly associated with the occurrence of rupture. Furthermore, the presence of coarse calcification inside the lymph nodes was associated with a favourable prognosis.

All the observed differences between the two groups were found to be statistically significant with a *P* value less than 0.05.

### Binary logistic multivariate regression analysis

In the binary logistic multivariate regression analysis (Table 1), the ultrasound signs that demonstrated statistically significant differences in the univariate analysis were included. The results of the multivariate regression analysis indicated that several ultrasound characteristics were independent risk indicators of CTL rupture. Specifically, a non-enhanced area in CEUS greater than 1/2, heterogeneous internal echotexture, perinodal echogenicity changed, and unclear margin of the lymph node were identified as independent risk signs of cervical lymph node tuberculosis rupture. On the other hand, the presence of coarse calcification inside the lymph node was found to be a sign that the lymph node is less likely to rupture. A logistic regression model was constructed to predict cervical lymph node tuberculosis rupture using ultrasound multimodality. The model is represented as follows: *Logistic* (*Y*) = −4.468 + 0.811 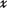 _1_ + 1.427 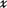 _2_ + 2.220 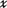 _3_ − 3.691 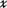 _4_ + 1.815 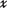 _5_ + 3.608 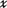 _6_.

The odds ratios (ORs) associated with each ultrasound sign, ranked from largest to smallest in absolute value, are as follows: coarse calcification in the lymph node ( 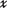 _4_) > non-enhanced area in CEUS greater than 1/2 ( 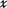 _6_) > heterogeneous internal echotexture ( 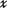 _3_) > perinodal echogenicity changed ( 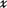 _5_) > unclear margin of the lymph node ( 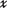 _2_) > long-to-short-axis ratio less than 2 ( 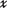 _1_).

### Establishment of a risk prediction and evaluation system for lymph node tuberculosis rupture

**Figure 2.**
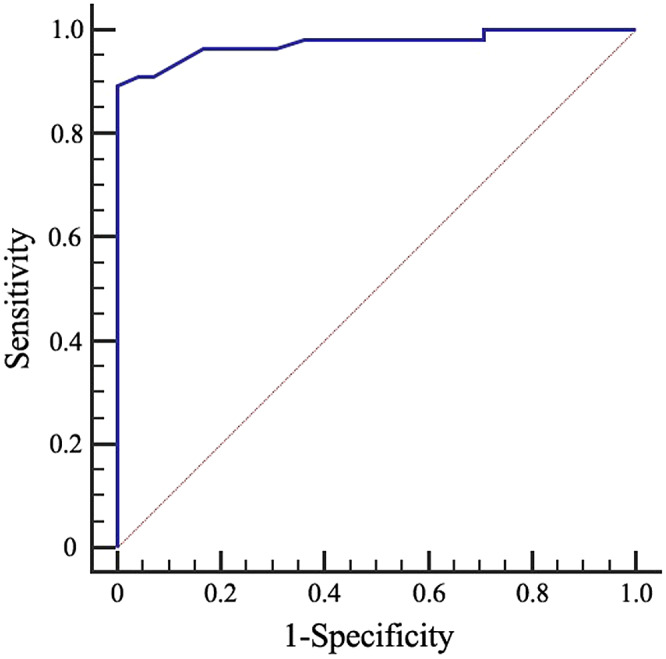
Receiver operating characteristic (ROC) curves for the diagnostic accuracy of the prediction model in cervical tuberculous lymphadenitis (CTL).

The logistic regression equation was utilized to assess the risk of TB rupture in cervical lymph nodes. Based on the collapse prediction integral of the characteristic signs, a ROC curve was plotted (Figure 2). The area under the curve (AUC) was determined to be 0.975, indicating that the model’s overall prediction accuracy reached 97.5%. The standard error was 0.0145, and the result was statistically significant with a *P* value of less than 0.001. The 95% CI for the AUC ranged from 0.930 to 0.994.

Using Youden’s index, the largest value (0.8929) was associated with a corresponding regression equation value of *Y* = −0.049. By incorporating the characteristic ultrasound indicators of tuberculous lymphadenitis cases into the regression equation, the *Y* value could be calculated. When *Y* ≥ −0.049, it was predicted that the lymph node would rupture, whereas when *Y* < −0.049, it was predicted that the lymph node would not rupture. This prediction approach exhibited a sensitivity of 89.29%, specificity of 100%, accuracy of 95.31%, positive predictive value of 100%, and negative predictive value of 92.31%.

## Discussion

The neck is the most common site for tuberculous lymphadenitis, which is the most common form of extrapulmonary TB [11]. Tuberculous lymphadenitis in the neck is typically caused by *Mycobacterium* tuberculosis accumulating in the nasal and oral cavities, resulting in submucosal infection along lymphatic vessels or spreading from adjacent pulmonary TB through lymphatic vessels to cervical lymph nodes [12]. This process is accompanied by an immune response and allergy (type IV) along with caseous necrosis, as the body attempts to destroy and eliminate *Mycobacterium* tuberculosis. Sensitized individuals often mount a faster defence response compared to unsensitized individuals, but tissue necrosis is also more pronounced, leading to different responses in the body.

**Figure 3.**
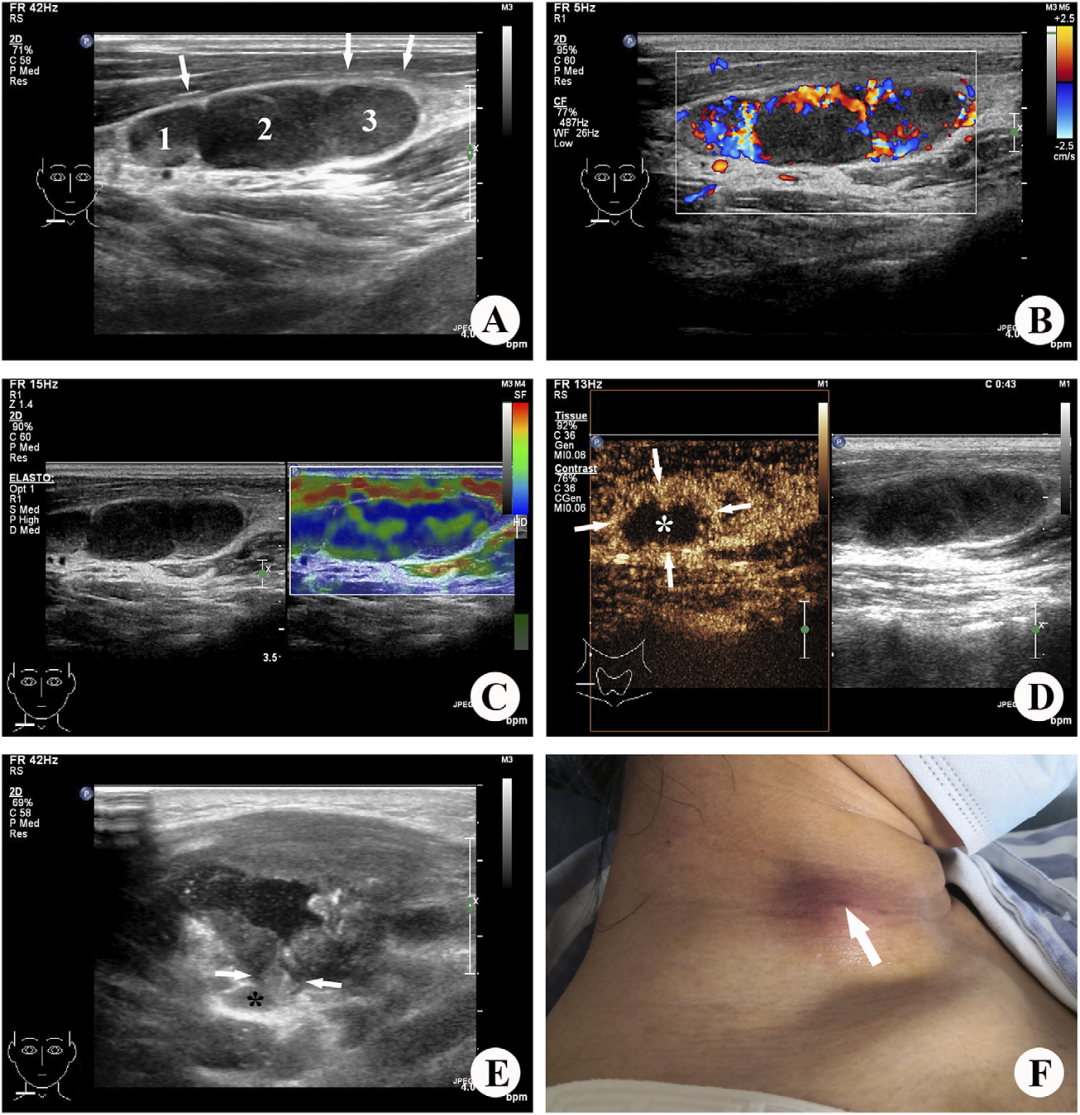
Images illustrating tuberculous lymphadenitis in a 31-year-old female patient, localized to the right side of the neck. (a) Grey-scale ultrasound revealed the presence of three enlarged lymph nodes exhibiting unclear margins and heterogeneous internal echotexture. Among them, the largest lymph node (2) measured approximately 1.2 × 0.8 cm (long-to-short-axis ratio (L/S) <2), with concurrent changes observed in the perinodal (muscular layer) echogenicity (→). (b) Colour Doppler ultrasound identified a limited number of colour signals indicative of blood flow within and surrounding the affected lymph node lesion. (c) Elastic ultrasound demonstrated the lymph nodes appearing as regions of blue and green, with an elastography score of 3. (d) Contrast-enhanced ultrasound (CEUS) imaging exhibited internal heterogeneous enhancement (→), with a non-enhanced area (*) encompassing more than half of the lesion. (e) Three months after the diagnosis of tuberculous lymphadenitis, the lymph node (*) ruptured into the superficial muscle layer through a fissure (→), resulting in pronounced muscle swelling. (f) Four months following the diagnosis, a small sinus formation became evident on the skin of the right neck.

With the development of technology, high-frequency ultrasound has become the preferred diagnostic tool for cervical lymph node diseases due to its real-time imaging, convenience, and non-radiation properties [13]. Additionally, elasticity ultrasound and CEUS are widely used for the auxiliary diagnosis of superficial masses [14]. Elasticity ultrasound allows for better evaluation of the softness and hardness of lesions, while CEUS provides an effective assessment of tissue perfusion and microcirculation in lesions [15]. In the case of lymph node tuberculosis, CEUS has shown diagnostic value [16–18], with findings of heterogeneous enhancement within lymph nodes and rim-like enhancement around the lesion [19]. Therefore, in spite of sonography is more heavily burdened than other imaging methods by subjective evaluation by the investigator,the integration of ultrasound multimodality, including elastography and CEUS, is expected to provide a comprehensive evaluation of lymph node lesions and contribute to the prediction of CTL rupture (Figure 3).

It has been widely accepted that the development process of lymph node tuberculosis is commonly divided into four stages [20, 21]. In the first stage, hyperplasia with granuloma formation occurs in the lymph node, leading to swollen, hardened, moderately active, and mildly tender, just after *Mycobacterium* tuberculosis infects the lymph nodes and lymphocytes and mononuclear cells proliferate to form granulomas. Ultrasound imaging during this stage often shows enlarged lymph nodes with reduced echogenicity and uniform enhancement on CEUS, which have no characteristic features on ultrasound images. In the second stage, caseous necrosis occurs within the lymph nodes, resulting in the destruction of the lymphatic hilum structure and composition of epithelioid cells, lymphocytes, Langerhans cells, and fibrous tissue around the caseous necrosis, which showed unclear internal structure on ultrasound. Non-enhanced areas within the lymph nodes and internal heterogeneous enhancement are characteristic manifestations of lymph node tuberculosis on CEUS [19]. The third stage involves the destruction of the lymph node capsule, leading to peri-lymphadenitis and lymph node fusion. At this stage, lymph node activity is limited, mainly tuberculous granulomas in the nodal and extranodal and chronic non-specific inflammatory reactions. Ultrasound imaging during this stage shows unclear boundaries and changes in echogenicity in the surrounding soft tissue layer. In the fourth stage, caseous necrosis in the lymph node combined with tissue liquefaction forms a TB abscess. The abscess may penetrate the invaded capsule, enter the subcutaneous or relatively loose surrounding soft tissue, form an abscess cavity, and eventually form a sinus tract.

The progression of lymph node tuberculosis is associated with increased necrosis within the lymph nodes, elevated internal pressure, and inflammatory reactions in the surrounding tissues. Ultrasound findings such as non-enhanced areas >1/2 on CEUS, lymph node L/S <2, unclear lymph node margins, and perinodal echogenicity changes are indicative of disease progression and are associated with a risk of rupture and sinus tract formation. In this study, these ultrasound manifestations were more frequently observed in the ruptured group compared to the unruptured group, with statistically significant differences (*P* < 0.05). The regression analysis also revealed that the absolute value of OR for the non-enhanced area >1/2 on CEUS was the largest, followed by heterogeneous internal echotexture, perinodal echogenicity changed, unclear margin of the lymph node, and lymph node L/S <2. Furthermore, the lesion volume in the ruptured group was significantly larger than that in the unruptured group, which may be related to internal pressure of the lymph node and infection severity. These findings are consistent with the disease progression.

The presence of coarse calcifications in lymph nodes is regarded as a distinctive manifestation of lymph node tuberculosis [22, 23]. The larger caseous necrosis within the lymph node is encompassed by the proliferation of fibrous tissue surrounding the lesion. The necrosis becomes concentrated and desiccated, accompanied by the deposition of calcium salts, resulting in the formation of substantial calcified foci. This process represents the healing stage of lymph node tuberculosis, clinically referred to as the induration calcification stage [23–25]. Following the encapsulation, concentration, and absorption of a significant number of abscesses and necrotic tissue within the lymph nodes, the pressure within the abscess cavity is diminished, thereby substantially reducing the risk of rupture. Therefore, the appearance of extensive calcifications in the affected lymph nodes signifies a healing outcome in lymph node tuberculosis. Consequently, lymph nodes are less susceptible to rupture at this time, which is consistent with the findings of this investigation. Among the 33 patients with pronounced calcified lymph nodes in this study, only one case experienced rupture. The extent of lymph node calcification in the ruptured group was significantly lower than that in the unruptured group, and this disparity was statistically significant (*P* < 0.05). Regression analysis revealed that the absolute value of the OR for coarse calcification in the lymph node was the largest and most negative, indicating that ultrasound appearance holds considerable predictive value for lymph node tuberculosis rupture.

Several studies have indicated that, except during the initial stage characterized by lymph node hyperplasia and granuloma formation, the colour blood flow signal may increase due to inflammatory stimulation in the development of lymph node tuberculosis [26, 27]. Therefore, it becomes challenging to assess the stage of lymph node tuberculosis lesions based on colour flow signals, thereby impeding the prediction of rupture risk. Some studies have also attempted to identify characteristic manifestations of lymph node tuberculosis using elastographic ultrasound, revealing that most lymph node tuberculosis lesions have an elasticity score ranging from 2 to 3 points [28–30]. Moreover, elastography is susceptible to calcification and liquefaction of lesions, as well as the subjective judgement and manipulation techniques of the operator. In addition, we also observed that there was a certain relationship between the rupture of CTL and age in this study, which may be due to the more severe allergic reaction in younger patients [31].

Naturally, this study possesses certain limitations. The same patient can exhibit different stages of lymph node tuberculosis development, leading to overlapping and diverse ultrasound manifestations. The study employed a 1-year follow-up period, neglecting long-term follow-up for all cases, which resulted in cases with slow progression being overlooked. Consequently, there is a degree of error in the follow-up results of the patients. Furthermore, all the case data were collected during the patients’ initial hospital visit, excluding information on the disease course prior to admission. Some cases also experienced repeated disease and transitional stages during treatment at our hospital. Although ultrasound-guided needle biopsy, coupled with Xpert MTB/RIF examination, can enhance the diagnostic rate of the disease, the invasive nature of the procedure inevitably carries a certain risk of lymph node rupture. While cases of rupture within 1 month after a puncture were excluded as per the study design, there are still factors that can contribute to rupture resulting from the procedure. The analysis of lymph node tuberculosis rupture is a complex and long-term undertaking, necessitating further comprehensive and detailed investigation in the future.

## Conclusion

In conclusion, this study demonstrated the effectiveness of ultrasound multimodality in evaluating cervical lymph node tuberculosis. It also employed statistical methods to identify key characteristics, such as L/S <2, unclear margin, heterogeneous internal echotexture, perinodal echogenicity changed, and non-enhanced area in CEUS >1/2, which serve as distinctive signs of easy to rupture of CTL. Furthermore, the presence of coarse calcifications in lymph nodes is indicative of CTL that is less prone to rupture. These findings offer theoretical support for the utilization of multimodal ultrasonography in comprehensively evaluating the overall condition of CTL.

## Data Availability

All the data are presented in the manuscript; any raw data can be available by request to the first author (Email: zjuzhaodan@163.com).
